# Addressing the Negative Impact of Social Media on Body Image: An Online Randomized Controlled Pilot Trial

**DOI:** 10.1002/eat.24584

**Published:** 2025-10-28

**Authors:** Gritt Ladwig, Kristine Schönhals, Hannah L. Quittkat, Fanny Alexandra Dietel, Silja Vocks

**Affiliations:** ^1^ Department of Clinical Psychology and Psychotherapy, Institute of Psychology Osnabrück University Osnabrück Germany

**Keywords:** body image, eating disorders, intervention, randomized controlled pilot trial, social media

## Abstract

**Objective:**

Previous research has revealed negative effects of appearance‐related social media content, such as fitspiration, on body satisfaction. However, specific interventions to reduce these detrimental effects are scarce. Therefore, this randomized controlled pilot trial investigated the efficacy of the four‐week online intervention body image booster (BIBo), which aims to reduce the negative influence of social media on body image by addressing theoretically proposed underlying mechanisms.

**Method:**

*N* = 157 female participants with elevated eating disorder (ED) symptoms were randomly allocated to the BIBo training or a waitlist control condition (WLC). The final sample included *n* = 38 completers in the BIBo training group and *n* = 46 completers in the WLC. Before and after the four training sessions, ED symptoms and reactivity to fitspiration content were assessed, as well as body dissatisfaction, social comparison processes, and internalization of body ideals.

**Results:**

BIBo participants showed significant pre‐post reductions in ED symptoms (*d* = −0.72) and reactivity to fitspiration content (*d* = −0.58), while WLC participants showed no significant change. The same pattern of results emerged for upward social comparison (*d* = −0.59), appearance comparison on social media (*d* = −0.89), body dissatisfaction (*d* = −0.40), and internalization of a thin body ideal (*d* = −0.42). There were no pre‐post changes in internalization of muscularity or attractiveness ideals in either the BIBo or the WLC group.

**Discussion:**

Overall, the results suggest that BIBo is effective in altering pertinent outcome and mechanistic measures related to social media use and body image, demonstrating its therapeutic potential in the prevention and treatment of EDs.

**Summary**

- This randomized waitlist‐controlled pilot trial investigated the online intervention Body Image Booster (BIBo), addressing the negative influence of social media on body image in women with elevated eating disorder symptoms.
- The results included reduced eating disorder symptoms, reduced reactivity to appearance‐related social media content, and reduced upward comparison following the BIBo intervention.
- The positive effects of BIBo suggest that BIBo modules might be applied in eating disorder treatments.

## 1 Introduction

Social media is omnipresent and tremendously popular, with global user numbers exceeding 5.2 billion in 2025 (Statista 2025). Yet, research also indicates that social media constitutes an important factor influencing body dissatisfaction (Roberts et al. 2022). This influence may be explained by the tripartite influence model (Roberts et al. 2022; Thompson et al. 1999), which posits that media, parents, and peers influence body dissatisfaction through social comparison and internalization of body ideals.

Empirically, findings on the proposed mediating role of ideal internalization remain mixed (Jarman et al. 2021; Jung et al. 2022; de Valle et al. 2021); however, social comparison has consistently been found to mediate the association between social media influences and body image (de Valle et al. 2021). With research showing that appearance‐focused social media use is particularly linked to body image concerns (Cohen et al. 2017), prior studies have found that exposure to so‐called “fitspiration” content is associated with increased appearance comparison and body dissatisfaction (Jeronimo and Carraca 2022). Such fitspiration content, typically aimed at women, features toned, thin bodies promoting a healthy lifestyle (Tiggemann and Zaccardo 2018). Although this thin‐muscular ideal is viewed as most attractive and preferred over a solely thin ideal (Bozsik et al. 2018), it is almost unachievable for most women (Tiggemann and Zaccardo 2018) and can exert harmful effects on body satisfaction (Prichard et al. 2020; Rounds and Stutts 2021). To date, various studies have underscored the negative impact of social media (e.g., Fardouly and Vartanian 2016) and of fitspiration content (e.g., Jeronimo and Carraca 2022; Ladwig et al. 2024; Rounds and Stutts 2021) on body image. However, few efforts have been undertaken to develop interventions protecting women from these effects.

Among these few extant interventions, most have focused on media literacy. Concerning traditional media, Bennett et al. (2023) found increased state body satisfaction in female undergraduates following a smartphone‐based intervention. Furthermore, Halliwell et al. (2011) showed that negative influences of thin‐ideal images on body satisfaction in adolescent girls could be prevented if participants watched an awareness video on image editing prior to image exposure. Relatedly, Arendt et al. (2017) demonstrated that watching an awareness video versus an active control condition, led to decreased appearance comparison. Notably, it appears crucial that such awareness material leverages appearance comparison (see de Valle et al. 2021) over strengthening body image alone, given prior findings showing that women with high and low body appreciation (Dignard and Jarry 2021), and women with and without self‐reported eating disorders (EDs; Ladwig et al. 2024) respond equally negatively to fitspiration. Consequently, devising interventions that address mediating influences on body dissatisfaction, that is, appearance comparison and body ideal internalization (Roberts et al. 2022), as per the tripartite influence model (Thompson et al. 1999), appears promising to enhance intervention efficacy.

Consistently, Tobin et al. (2022) found that participation in a media literacy intervention and participation in a cognitive dissonance‐based intervention (encouraging participants to argue against the persuasion of a thin body ideal) versus waitlist, led to stronger reductions in body dissatisfaction, eating disorder symptoms, and thin ideal internalization. In a social media context, Bell et al. (2022) found increased body satisfaction and reduced thin‐ideal internalization in adolescents after a combined dissonance‐based and media literacy classroom intervention. Further research in this realm has underscored the positive effects of media literacy interventions on body‐related measures, for example, concerning reduced dietary restraint in adolescent girls (Gordon et al. 2021; McLean et al. 2017). Beyond (social) media literacy interventions, classroom‐based programs for adolescents have been developed, combining skills to deal with appearance‐related conversations and body‐related comparisons (Bird et al. 2013) or body image‐related psychoeducation and “lifestyle” factors (Svantorp‐Tveiten et al. 2021) with (social) media literacy interventions. Such interventions showed beneficial effects on body satisfaction, appearance comparison and eating behaviors (Bird et al. 2013), as well as thin ideal internalization in girls (Svantorp‐Tveiten et al. 2021). Two further studies investigated non‐literacy‐based approaches (i.e., a self‐guided cognitive‐behavioral program to reduce self‐criticism in university students, de Valle and Wade 2022; and a self‐compassion writing task intervention, Gobin et al. 2022), demonstrating post‐treatment reductions in appearance comparison, appearance‐related social media motivation, and disordered eating (de Valle and Wade 2022), and reduced appearance and weight dissatisfaction (Gobin et al. 2022).

In sum, to date, only a few studies have harnessed appearance comparison and body ideal internalization as the proposed mechanisms underlying detrimental appearance‐related social media effects (Roberts et al. 2022). Further, of note, all aforementioned studies, except one (Tobin et al. 2022), examined healthy adolescents or women without elevated body dissatisfaction or EDs (Bell et al. 2022; Bennett et al. 2023; Bird et al. 2013; de Valle and Wade 2022; Gobin et al. 2022; McLean et al. 2017; Svantorp‐Tveiten et al. 2021). Thus, it remains unclear whether women with elevated ED symptoms would respond similarly to these interventions. Importantly, this group warrants particular attention as they are more likely to view appearance‐related social media content, including fitspiration (Christensen Pacella et al. 2023; Griffiths et al. 2024).

To address this gap, we developed *Body Image Booster* (BIBo), a 4‐week online group intervention for women with elevated ED symptoms that builds on the effectiveness and enhanced accessibility of online interventions in reducing ED symptoms (Aardoom et al. 2016; Melioli et al. 2016; Taylor et al. 2021). The present study comprises a randomized controlled pilot trial (RCT) to investigate the efficacy of BIBo versus a time‐equivalent waitlist control condition (WLC). Consistent with the tripartite model (Roberts et al. 2022; Thompson et al. 1999) and empirical evidence, we predicted that participants undergoing BIBo versus the WLC, would show stronger pre–post reductions in (1) reactivity to appearance‐related social media content, that is, fitspiration imagery, and (2) eating disorder symptoms (primary endpoints). Furthermore, we predicted that BIBo versus WLC, participants would show stronger pre–post reductions in (3) body dissatisfaction, (4) social comparison (i.e., upward social comparison and comparison on social media), and (5) internalization of body ideals (i.e., thin, attractive, and muscular body ideal; secondary endpoints).

## 2 Method

### 2.1 Trial Design

This two‐arm RCT comprised a screening and two assessment points (i.e., pre‐ and post‐assessment) and was conducted between March and November 2024 at Osnabrück University. Randomized condition allocation was based on a random number generator implemented via the survey platform LimeSurvey (version 6.5, 2024). This study was approved by the ethics committee of Osnabrück University. Study reporting follows the Consolidated Standards for Reporting Trials [CONSORT] statement for randomized controlled trials (Hopewell et al. 2025).

### 2.2 Participants

Participants were recruited via social media, mailing lists, and press releases. Inclusion criteria were a minimum age of 16 years, female gender, regular use of the social media platforms Instagram, Facebook, and/or TikTok, and ED symptoms (i.e., total score ≥ 15 on the Eating Disorder Examination Questionnaire–Short (*EDE‐QS*); Gideon et al. 2016, 2018, as an EDE‐QS score of 15 has been established as the best possible cut‐off to identify probable ED cases; Prnjak et al. 2020). Exclusion criteria were a body mass index (BMI) < 14, diagnosis of substance abuse or dependency, psychosis, borderline personality disorder, and self‐harming behaviors including suicidality. The complete study eligibility criteria were displayed at the beginning of the online screening. Participants were reimbursed with vouchers worth 30 Euros.

**FIGURE 1:**
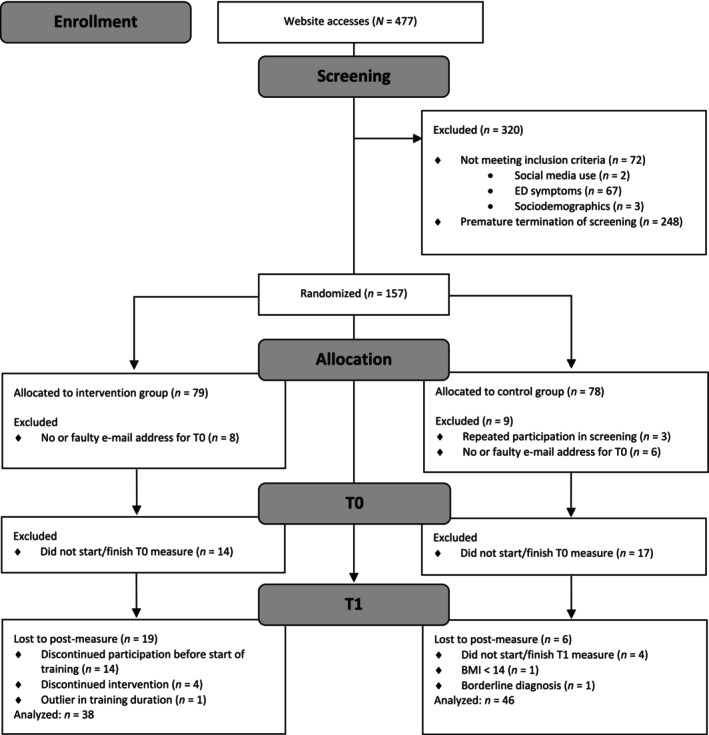
CONSORT flow‐chart.

Of *N* = 477 participants who accessed the study link, *n =* 157 were randomized and *n* = 84 were included in the completer analysis (see Figure 1). Table 1 provides sample characteristics.

**TABLE 1:** Baseline demographics.

	BIBo (*n* = 38) M (SD)/*n* (%)	WLC (*n* = 46) M (SD)/*n* (%)	Total (*N =* 84) M (SD)/*n* (%)
Age (years)	25.76 (5.84)	28.83 (7.76)	27.44 (7.09)
Body mass index (kg/m^2^)	22.42 (4.63)	23.14 (6.51)	22.83 (5.76)
Sexual orientation			
Heterosexual	58.82%	75.00%	67.95%
Homosexual	2.94%	0.00%	1.28%
Bisexual	35.29%	18.18%	25.64%
Other	0.00%	0.00%	0.00%
Daily social media use^a^			
TikTok	2.29 (0.91)	2.00 (1.08)	2.15 (0.99)
Instagram	2.54 (0.93)	2.67 (1.10)	2.61 (1.02)
Facebook	1.38 (0.52)	1.60 (0.63)	1.52 (0.59)
Snapchat	1.28 (0.46)	1.39 (0.78)	1.33 (0.63)
BeReal	1.00 (0.00)	1.14 (0.38)	1.07 (0.26)
Weekly social media use^b^			
TikTok	3.64 (0.84)	3.46 (0.88)	3.56 (0.85)
Instagram	3.78 (0.58)	4.00 (0.00)	3.90 (0.40)
Facebook	3.00 (1.20)	3.27 (0.88)	3.17 (0.98)
Snapchat	3.44 (1.04)	3.83 (0.51)	3.64 (0.83)
BeReal	3.13 (1.13)	4.00 (0.00)	3.53 (0.92)
Mental disorder			
Current diagnosis	68.42%	73.91%	71.43%
Current treatment	92.31%	73.53%	81.67%
Current diagnosis and current treatment	63.16%	54.35%	58.34%
Treatment addressing appearance‐related social media use	25.00%	20.00%	22.45%
Highest educational attainment			
No qualifications	0.00%	0.00%	0.00%
Lower‐track secondary school	0.00%	0.00%	0.00%
Medium‐track secondary school	7.89%	4.35%	5.95%
Vocational higher‐track secondary school	5.26%	10.87%	8.33%
Higher‐track secondary school	50.00%	36.96%	42.86%
Vocational college degree	10.53%	10.87%	10.71%
University degree	26.32%	36.96%	32.14%

Abbreviations: BIBo = Body Image Booster; M = mean; SD = standard deviation; WLC = Waitlist Control.

^a^ Means and standard deviations are based on a 6‐point Likert scale with the following anchors: 1 = “less than 1 hour”, 2 = “1 to 2 hours”, 3 = “2 to 3 hours”, 4 = “3 to 4 hours”, 5 = “4 to 5 hours”, 6 = “more than 5 hours”.

^b^ Means and standard deviations are based on a 4‐point Likert scale with the following anchors: 1 = “1 day”, 2 = “2 to 3 days”, 3 = “4 to 5 days”, 4 = “6 to 7 days”.

### 2.3 Measures^1^

Unless otherwise specified, higher scores on the scales described below indicate higher symptom severity. For information on psychometric properties, including internal consistency, of the applied questionnaires, see Supporting Information.

#### 2.3.1 Primary Endpoints

##### 2.3.1.1 Reactivity to Appearance‐Related Social Media Content

To assess state reactions to fitspiration images, participants completed a free‐viewing task (Ladwig et al. 2024). The applied images were sourced from Instagram by conducting a search using the hashtags #fitspo and #fitspiration. Overall, posts showed a slender, muscular female body in different poses, embodying the fitspiration trend, without prominent tattoos. We used *n* = 30 original Instagram posts (i.e., photo and caption) from content creators who provided written informed consent. Before and after image presentation, participants completed the Body Image States Scale (BISS, Cash et al. 2002; Vocks et al. 2007), a six‐item questionnaire measuring state body dissatisfaction.

##### 2.3.1.2 ED Symptoms

To assess ED symptom severity within the last week, we used the 12‐item *Eating Disorder Examination Questionnaire–Short (EDE‐QS*, Gideon et al. 2016, 2018; own translation).

#### 2.3.2 Secondary Endpoints

##### 2.3.2.1 Body Dissatisfaction

To measure body dissatisfaction, we employed the nine‐item *Body Dissatisfaction* subscale of the *Eating Disorder Inventory‐2* (*EDI‐2*, Garner 1991; Paul and Thiel 2005).

##### 2.3.2.2 Social Comparison

To assess social comparison with subjectively perceived more attractive individuals, we used the *Upward Appearance Comparison Scale (UPACS*; O'Brien et al. 2009; Schönhals et al. 2024).

To measure processes and consequences of appearance comparison on social media, we employed the *Appearance Comparison on Social Media Scale* (*ACSMS*, Mahon and Hevey 2023; own translation).

##### 2.3.2.3 Internalization of Appearance Ideals

To measure body ideal internalization, we used the subscales “Internalization: Muscular”, “Internalization: General Attractiveness”, and “Internalization: Thin/Low Body Fat” from the *Sociocultural Attitudes Towards Appearance Questionnaire‐4‐Revised* (*SATAQ‐4R‐Female*, Schaefer et al. 2017; Flechsig et al. 2025).

#### 2.3.3 Additional Measures

To assess sample characteristics and eligibility criteria, participants provided sociodemographic and mental health‐related information, that is: age, weight, height, gender, social media consumption, highest educational attainment, sexual orientation, and presence of any mental health issues. If applicable, participants were asked whether they were receiving mental health treatment and if so, whether their treatment included information on the influence of social media on body image. To obtain immediate feedback on the acceptability of the intervention, participants could optionally and anonymously rate the statement “I now feel more able to cope with the influences of appearance‐related social media than before” on a scale from 0 (*not at all*) to 6 (*exactly*) after each session.

### 2.4 Conditions

#### 2.4.1 Body Image Booster (BIBo)

The BIBo intervention comprised four modules that were scheduled as weekly, ~60‐min online group sessions with two to seven participants. Modules one through three included homework assignments that were non‐mandatory to keep the intervention low threshold. Sessions were led by a licensed psychotherapist or a psychologist in advanced training, using the software BigBlueButton (version 2.5, 2022). The software was operated on servers of Osnabrück University, complying with European data protection regulations. BIBo builds on established interventions to reduce ideal internalization and appearance comparison, that is, psychoeducation, dissonance‐based interventions (Stice et al. 2000; Tobin et al. 2022), and media literacy interventions (e.g., Arendt et al. 2017; Svantorp‐Tveiten et al. 2021). Importantly, BIBo focuses on the social media context, including strategies to cope with fitspiration exposure as well as metacognitive techniques to reduce rumination (Hjemdal et al. 2019; Wells 2008) after social media use, since rumination bears the risk of increasing body dissatisfaction (Rivière et al. 2018). Moreover, strategies focusing on body functionality to promote body satisfaction (e.g., Alleva et al. 2015; Smith et al. 2023) and techniques to draw attention away from perceived physical flaws are included. Our stimulus material entailed self‐drawn illustrations of cartoon characters with different body sizes, and illustrated videos on body image, social comparisons, and body neutrality/body functionality created via the software Powtoon (https://www.powtoon.com, 2023). Module presentations were created using PowerPoint (Microsoft Office Standard 2019, version 1809). Table 2 shows detailed module descriptions.

**TABLE 2:** Module description of BIBo sessions.

BIBo session	Main contents	References
Session 1: Body image	*Psychoeducation and media literacy* Early influences of the media on body image (e.g., TV characters, dolls) Information video on the development and maintenance of body dissatisfaction Discussion of individual factors influencing body dissatisfaction Presentation of research findings on double standards and dysfunctional attentional focus in women *Exercise: Focus of attention* Shifting the focus of attention from negative self‐evaluation to external factors when viewing images of oneself *Voluntary homework* Practicing attention shifts in everyday life	Jansen et al. (2005) Legenbauer and Vocks (2014) Veale and Neziroglu (2010) Voges et al. (2018) Voges et al. (2022) Wilhelm et al. (2014)
Session 2: Social comparison	*Awareness video* *Exercise: Media literacy* Detecting edited images on social media Group discussion on exercise results *Information video on social comparison* *Metacognitive techniques* Airport metaphor (i.e., letting go of negative thoughts) Defusion techniques (e.g., distancing oneself from negative thoughts by mentally assigning a funny voice to them) *Voluntary homework* Practicing metacognitive techniques	Arendt et al. (2017) Bennett et al. (2023) Dambacher and Samaan (2020) Exner and Hansmeier (2020) Halliwell et al. (2011) Tobin et al. (2022) Wengenroth (2017)
Session 3: Body neutrality	*Psychoeducation on self‐esteem* *Exercise: self‐esteem pie chart* Sources of self‐esteem and the individual role of weight, figure, and appearance Discussion of the results in small groups *Psychoeducation* Information video on Body Neutrality and Body Functionality *Exercise: Body Functionality* Writing task (e.g., writing down how the body helps us in everyday life and how its functions enable us to have positive experiences) *Voluntary homework* Summary of the writing exercise	Alleva et al. (2015) Fehm and Weidmann (2023) Guest et al. (2019) Potreck (2014)
Session 4: questioning body ideals	*Exercise: risks and benefits of pursuing body ideals* Discussion of the results in small groups *Exercise: Induction of cognitive dissonance* “Fat Talk” I—criticizing ideals “Fat Talk” II—active implementation of arguments in role play *Exercise: Focus of attention (see session 1)* Shifting the focus of attention from negative self‐evaluation to external factors when viewing images of oneself	Bird et al. (2013) Legenbauer and Vocks (2014) Stice et al. (2000) Stice et al. (2012) Stice et al. (2015)

#### 2.4.2 Waitlist Control (WLC) Condition

WLC participants underwent a 5‐week (i.e., time‐equivalent) waiting period before receiving the BIBo intervention.

### 2.5 Procedure

**FIGURE 2:**
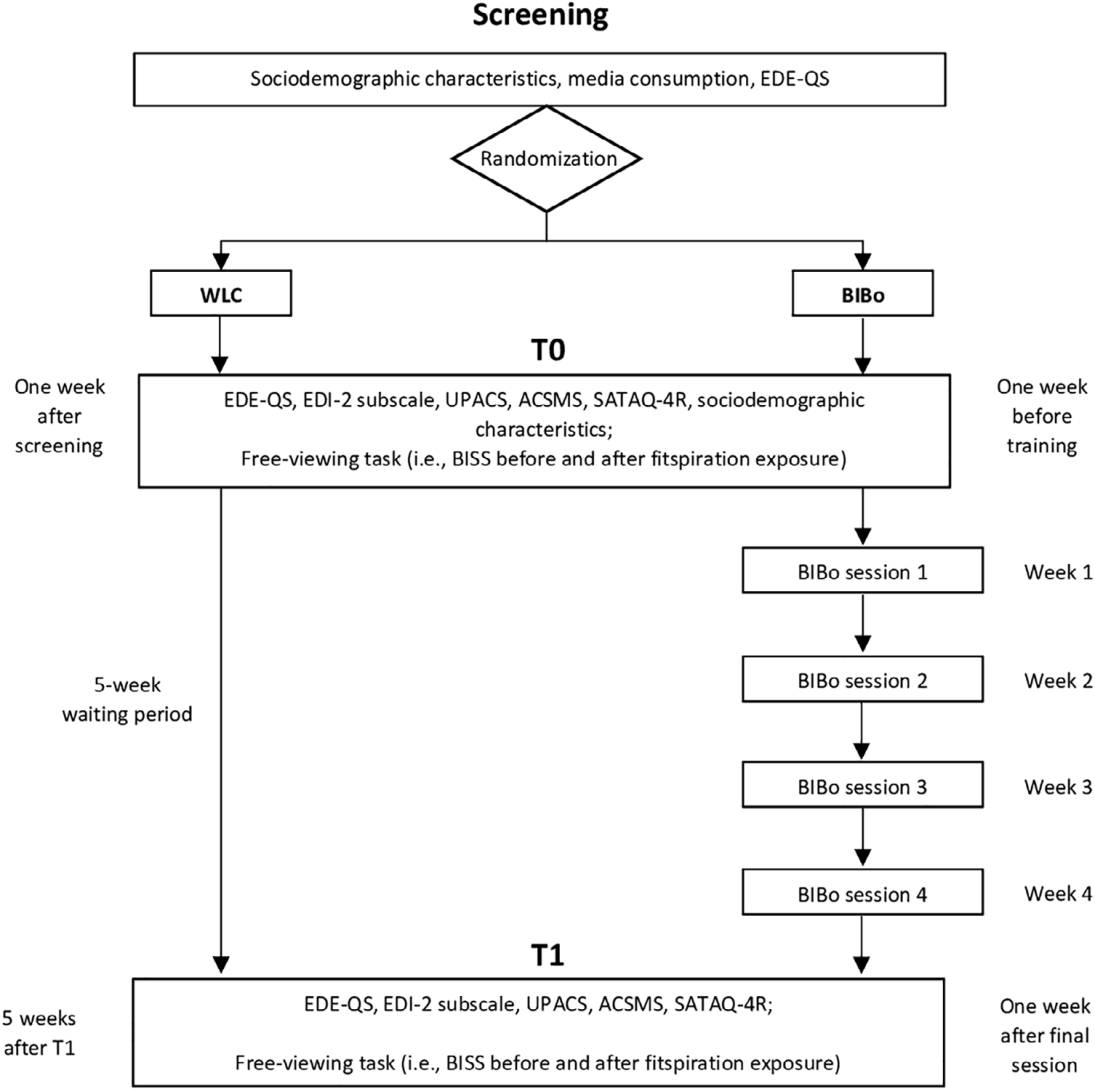
Study procedure. ACSMS = Appearance Comparison on Social Media Scale; BISS = Body Image States Scale; EDE‐QS = Eating Disorder Examination Questionnaire—Short; EDI‐2 = Eating Disorder Inventory‐2; SATAQ‐4R = Sociocultural Attitudes Towards Appearance Questionnaire‐4‐Revised; UPACS = Upward Appearance Comparison Scale.

Participants accessed the online screening, whereupon they received information on the study procedure, eligibility criteria, data processing, and required confidentiality concerning private information disclosed during group sessions. After providing informed consent, participants completed screening measures to assess study eligibility. Ineligible participants received contact information for mental health support centers if needed. Participants were then randomized to BIBo or WLC. One week before training commenced (BIBo) or 1 week after screening (WLC), participants completed the pre‐assessment (T0). Following T0, participants in the BIBo condition received the four training modules over 4 weeks, while WLC participants underwent a five‐week waiting period, during which they were asked to use social media as usual. After this waiting period (WLC) or 1 week after the final module (BIBo), participants completed the post‐assessment (T1). For ethical reasons, WLC participants were offered the training after completing T1. Figure 2 shows the study procedure.

### 2.6 Statistical Analyses

Analyses were conducted in accordance with previous RCT rationales (e.g., Dietel et al. 2024). Specifically, we performed completer analysis and intention‐to‐treat analysis (ITT). For completer analysis, we included participants with complete datasets up to T1 (*n* = 84). For ITT, we included randomized participants (*n* = 157). To test the assumption that data were missing completely at random, we compared sociodemographic and psychometric baseline scores across dropout and completer samples using *t*‐tests. Total scores were calculated for self‐report outcomes. To operationalize reactivity to the free‐viewing task, we further computed difference scores for total BISS values (after—before task).

To explore between‐condition baseline differences, we conducted independent *t*‐tests for continuous variables and *χ* ^2^ tests for categorical variables. To investigate hypotheses, we computed linear mixed models (LMM) using the “lme4” package (Bates et al. 2015) in R (R Core Team 2024). LMM offer advantages for intervention data as they are superior in handling nested and missing data while relaxing restrictive assumptions, thereby enhancing statistical power (Hilbert et al. 2019).

LMM included *time* (i.e., time points) and *group* (i.e., BIBo, WLC) as predictors. Restricted maximum likelihood (REML) estimation was used for model fitting to ensure unbiased variance estimates. To investigate interactions, we computed pairwise group contrasts per time point and group comparisons over time, including 95% confidence intervals (CIs), using the “emmeans” package (Lenth et al. 2018). As a measure of effect size, Cohen's *d* was computed using raw scores, with paired‐sample *d* for within‐group changes and unpaired *d* for between‐group differences at T1. To ensure comparability of the results with other studies, we additionally performed 2 × 2 mixed analyses of variance (ANOVAs) per outcome, with the within‐subject factor *time* (i.e., time points) and the between‐subject factor *group* (i.e., BIBo or WLC), including follow‐up pairwise group contrasts per time point and group comparisons over time.

Tests were two‐tailed, with a significance threshold of *p <* 0.05. For multiple comparisons, Bonferroni correction was applied by adjusting *p*‐values according to the number of tests performed.

## 3 Results

### 3.1 Participants

Sociodemographic and psychometric sample characteristics are shown in Tables 1 and 3. Following Bonferroni correction, there were no significant between‐condition baseline differences across variables (all *ps* > 0.01).

### 3.2 Session Compliance and Dropout

*N* = 25 dropouts (i.e., *n* = 19 [33.33%] in BIBo, *n* = 6 [11.54%] in WLC) from T0 to T1 were recorded after the training or waiting period had commenced (see Figure 1). Importantly, following Bonferroni correction, dropouts and completers did not differ across conditions regarding sociodemographic and psychometric baseline variables (all *ps* > 0.02), corroborating the plausibility of dropouts missing at random.

### 3.3 Completer Analysis^2^

Tables 3 and 4 show means and SD across time points for self‐report outcome measures and the BISS before and after the free‐viewing task (i.e., reactivity to fitspiration), respectively.

**TABLE 3:** Effects of conditions on trait‐like psychometric measures.

	T0		T1	
	BIBo M (SD)	WLC M (SD)	BIBo M (SD)	WLC M (SD)
EDE‐QS	21.42 (6.24)	20.09 (4.52)	16.74 (6.76)	19.83 (5.05)
UPACS	4.23 (0.48)	4.05 (0.57)	3.84 (0.71)	4.05 (0.55)
EDI2 BD	44.55 (7.65)	42.43 (6.93)	41.55 (7.39)	42.63 (7.56)
ACSMS	24.08 (5.66)	23.46 (5.53)	19.39 (4.70)	22.93 (6.73)
SATAQ Musc	3.58 (1.16)	3.49 (1.10)	3.44 (1.15)	3.50 (1.09)
SATAQ Attr	4.67 (0.46)	4.49 (0.44)	4.43 (0.53)	4.41 (0.59)
SATAQ Thin	4.41 (0.81)	3.93 (0.86)	4.05 (0.90)	3.98 (0.86)

Abbreviations: ACSMS = Appearance Comparison on Social Media Scale; BIBo = Body Image Booster; EDE‐QS = Eating Disorder Examination Questionnaire Short; EDI2 BD = Eating Disorder Inventory 2: Body Dissatisfaction; RSES = Rosenberg Self‐Esteem Scale; SATAQ Attr = Sociocultural Attitudes Towards Appearance Questionnaire Attractiveness; SATAQ Musc = Sociocultural Attitudes Towards Appearance Questionnaire Muscularity; SATAQ Thin = Sociocultural Attitudes Towards Appearance Questionnaire Thinness; UPACS = Upward Physical Appearance Comparison Scale; WLC = Waitlist Control.

**TABLE 4:** Effects of conditions on reactivity to social media content.

	T0				T1			
	Before free viewing		After free viewing		Before free viewing		After free viewing	
	BIBo M (SD)	WLC M (SD)	BIBo M (SD)	WLC M (SD)	BIBo M (SD)	WLC M (SD)	BIBo M (SD)	WLC M (SD)
BISS	6.88 (1.24)	6.72 (1.34)	7.65 (1.40)	7.43 (1.28)	5.92 (1.64)	6.68 (1.49)	6.18 (1.67)	7.29 (1.45)

Abbreviations: BIBo = Body Image Booster; BISS = Body Image States Scale; WLC = Waitlist Control.

### 3.4 Session Feedback

Voluntary session feedback was provided by participants in the BIBo group and WLC who were offered training participation after post‐assessment (see Table 5).

**TABLE 5:** Means and standard deviations of immediate session feedback.

	*n*	M (SD)
Session 1	62	3.63 (0.83)
Session 2	57	4.18 (0.97)
Session 3	46	4.26 (1.08)
Session 4	47	4.70 (0.86)

*Note*: Participants gave session feedback in response to the statement “I now feel more able to cope with the influences of appearance‐related social media than before” on a scale from 0 (*not at all*) to 6 (*exactly*).

Abbreviations: M = mean; *n* = sample size; SD = standard deviation.

### 3.5 Primary Outcome Measures

#### 3.5.1 Reactivity to Appearance‐Related Social Media Content

Regarding change scores on the BISS, we found a significant time × group interaction, *b* = 0.502, 95% CI [0.068, 0.935], *p* = 0.026, indicating pre‐post reductions in BIBo, *b* = −0.509, 95% CI [−0.835, −0.183], *p* = 0.003, *d* = −0.58, but not in the WLC, *b =* 0.007, 95% CI [−0.289, 0.303], *p* = 0.961, *d* = −0.01. No significant between‐group differences emerged at T1, *b* = −0.350, 95% CI [−0.728, 0.028], *p* = 0.069, *d* = −0.42.

#### 3.5.2 Eating Disorder Symptoms

Regarding the EDE‐QS, a significant time × group interaction emerged, *b* = 4.423, 95% CI [2.753, 6.094], *p* < 0.001, indicating a pre‐post reduction in BIBo, *b* = −4.684, 95% CI [−5.940, −3.430], *p* < 0.001, *d* = −0.72, while the WLC remained unchanged, *b* = 0.261, 95% CI [−0.880, 1.400], *p* = 0.651, *d* = −0.05. At T1, BIBo versus WLC, participants had significantly lower EDE‐QS scores, *b* = −3.090, 95% CI [−5.540, −0.642], *p* = 0.014, *d* = −0.53.

### 3.6 Secondary Outcome Measures

#### 3.6.1 Social Comparison

Regarding the UPACS, a significant time × group interaction emerged, *b* = 0.391, 95% CI [0.198, 0.584], *p* < 0.001, driven by a pre‐post reduction in BIBo, *b* = −0.387, 95% CI [−0.532, −0.242], *p* < 0.001, *d* = −0.59, whereas the WLC remained stable, *b* = −0.004, 95% CI [−0.136, 0.128], *p* = 0.948, *d* = 0.01. At T1, between‐group differences were non‐significant, *b* = −0.207, 95% CI [−0.461, 0.046], *p* = 0.107, *d* = −0.33.

#### 3.6.2 Appearance Comparison on Social Media

Regarding the ACSMS, a significant time × group interaction emerged, *b* = 4.163, 95% CI [2.372, 5.953], *p* < 0.001, indicating a pre‐post reduction in BIBo, *b* = −4.684, 95% CI [−6.030, −3.339], *p* < 0.001, *d* = −0.89, while the WLC remained unchanged, *b* = 0.522, 95% CI [−0.701, 1.740], *p* = 0.399, *d* = −0.08. At T1, BIBo versus WLC, participants showed significantly lower scores, *b* = −3.540, 95% CI [−6.040, −1.040], *p* = 0.006, *d* = −0.60.

#### 3.6.3 Body Dissatisfaction

Regarding the EDI‐2 body dissatisfaction subscale, we found a significant time × group interaction, *b* = 3.196, 95% CI [1.767, 4.624], *p* < 0.001, with a pre‐post decrease in BiBo, *b* = −3.000, 95% CI [−4.070, −1.930], *p* < 0.001, *d* = −0.40, and no change in the WLC, *b* = −0.196, 95% CI [−1.170, 0.780], *p* = 0.691, *d* = 0.03. At T1, between‐group differences were non‐significant, *b* = −1.080, 95% CI [−4.290, 2.130], *p* = 0.507, *d* = −0.14.

#### 3.6.4 Body Ideal Internalization (Thin/Low Body Fat, Muscularity, and General Attractiveness Ideal)

Considering the body ideal internalization subscales of the SATAQ‐4R, for the thin ideal, we found a significant time × group interaction, *b* = 0.411, 95% CI [0.176, 0.645], *p =* 0.001, driven by a pre‐post reduction in BIBo, *b* = −0.362, 95% CI [−0.535, −0.188], *p <* 0.001, *d* = −0.42, but not the WLC, *b* = 0.049, 95% CI [−0.111, 0.209], *p =* 0.545, *d* = 0.06. However, the between‐group contrast was non‐significant at T1, *b* = 0.068, 95% CI [−0.304, 0.440], *p =* 0.719, *d* = 0.08. For general attractiveness and muscularity, time × group interactions were non‐significant, that is, *b* = 0.156, 95% CI [−0.027, 0.340], *p =* 0.099, and *b* = 0.146, 95% CI [−0.074, 0.367], *p =* 0.196, indicating no differential group‐related changes over time.

### 3.7 Intention‐To‐Treat Analysis (ITT)

The result pattern was replicated in the ITT, including the conventional interpretation of effect sizes (see Supporting Information).

## 4 Discussion

Consistent with hypotheses, in this study, participants undergoing the BIBo intervention showed a reduction in reactivity to fitspiration images from pre‐ to post‐intervention, while reactivity in the WLC remained unchanged. This finding demonstrates that reactivity to potentially harmful social media content can be modified by a brief intervention. Similarly, a previous study reported that reactivity to fitspiration content may be mitigated by an affirmation writing task prior to fitspiration exposure (Pilot and Stutts 2023). Our findings hold promise for women with elevated ED symptoms, since more frequent fitspiration exposure has been related to higher ED symptom severity (Dondzilo et al. 2024; Griffiths et al. 2018), and mitigating reactivity to such content might buffer its impact. Critically, although BIBo did not address the reduction of ED symptoms directly, symptom severity nevertheless decreased considerably in the intervention group, with a medium to large effect size. This is noteworthy because BIBo constitutes a low‐threshold, easily accessible program of limited duration in an economically efficient setting. Although such effect sizes might not be fully comparable to those found in prior research due to differing study designs, our results are promising, as the moderate to large effects found in our pilot trial exceed those reported in most previous studies (e.g., Bell et al. 2022; McLean et al. 2017; Tobin et al. 2022).

Consistent with the adapted tripartite influence model (Roberts et al. 2022), it appears likely that the achieved reduction of ED symptoms can be attributed to a reduction in the proposed mediating processes, i.e., social comparison and internalization, in turn leading to reduced body dissatisfaction and fewer ED symptoms. Indeed, in line with our hypotheses, social comparison and body dissatisfaction both decreased in the BIBo condition. These findings are encouraging, since a positive body image (Dignard and Jarry 2021) or the absence of an ED diagnosis alone (Ladwig et al. 2024) does not seem to be sufficient to reduce harmful social media influences; hence, modifying central mediating variables seems to represent an auspicious approach. The demonstrated reduction in upward appearance comparison is especially important given that upward appearance comparison has repeatedly been shown to mediate the relationship between social media use and body image (de Valle et al. 2021). Furthermore, upward comparison can predict body dissatisfaction following fitspiration exposure, underlining its potential as a driver of harmful fitspiration effects (Ladwig et al. 2025). Concerning internalization, we found that thin ideal internalization, but not muscular or attractiveness ideal internalization, declined following BIBo. It is probable that dissonance‐based strategies included within BIBo account for this decrease in thin ideal internalization, as previous research has found beneficial effects of such strategies on thin ideal internalization (Stice et al. 2019). However, as we found no effects on muscular ideal internalization, it appears that BIBo does not sufficiently address this body ideal. To date, studies adapting dissonance‐based interventions to target muscular ideal internalization have focused on male body image (Brown et al. 2017) or female athletes (Hirsch et al. 2022; Smith and Petrie 2008). Although the evidence on the mediating role of ideal internalization is inconsistent (de Valle et al. 2021; Jarman et al. 2021; Jung et al. 2022), future research should consider incorporating such adapted dissonance‐based strategies within BIBo to address the increasing importance of muscularity in the female body ideal (Bozsik et al. 2018). However, lacking interventional effects of BIBo on muscular ideal and attractiveness ideal internalization might also be grounded in statistical power, as this pilot RCT was sufficiently powered to detect large but not medium or small *time × group* interaction effects (*1‐ β* = 48%). Thus, larger trials are needed to observe smaller‐sized effects.

Some further limitations warrant acknowledgement. First, for administrative reasons, the study design required randomization prior to pre‐assessment, which may have differentially influenced possible expectancy effects. Such expectancy effects cannot be ruled out, although it should be noted that there were no a priori differences between BIBo and the WLC, rendering it likely that the observed effects are attributable to our intervention. Furthermore, the lack of pre‐registration of the study constitutes a limitation. Second, to reduce participant burden, we did not evaluate individual session effects, thus precluding the ability to draw conclusions about module‐based effects. Future studies should therefore examine individual training components, for example, by presenting session‐based symptom‐oriented questionnaires. Concerning treatment acceptability, voluntary session feedback concerning the ability to cope with social media influences improved across sessions, fluctuating around the response “rather yes.” This potentially indicates that participants found the intervention helpful, although results have to be interpreted cautiously as voluntary participation in surveys bears the risk of a self‐selection bias (Stone et al. 2024). Future studies should hence implement mandatory session feedback. Beyond the session feedback, we did not evaluate treatment acceptability, which thus warrants future investigation. Relatedly, reasons for the substantial, albeit typical dropout rates in the active condition (33.33%; e.g., Linardon and Fuller‐Tyszkiewicz 2020) should be investigated in future research to prevent premature intervention termination. Third, the group setting may have exerted distinct effects, as group cohesion and treatment outcomes can be positively correlated (Burlingame et al. 2018). Future research might therefore devise BIBo variants for other settings, for example, individual face‐to‐face and blended care approaches to distinguish between unspecific group effects and specific training effects. Fourth, to achieve high ecological validity, we did not exclude participants with a current diagnosis of any mental disorder beyond EDs (for exceptions, see *Participants*) or participants receiving psychiatric/psychotherapeutic treatment, which may have influenced the pattern of results. However, it should be noted that only just over half of the participants in each condition (i.e., 63.16% in BIBo and 54.35% in WLC) reported a mental disorder and were concurrently receiving treatment in addition to BIBo, with a descriptive but non‐significant group difference (see Table 1). Hence, any differential effects in BIBo or WLC are likely attributable to condition effects. Nevertheless, future studies might investigate the type, duration, and dosage of concurrent treatments to control for a possible influence on BIBo outcomes. Lastly, based on suggestions for control conditions in mobile health (Goldberg et al. 2023), this pilot trial was designed as a waitlist‐controlled RCT. However, this limits the generalizability to other pertinent comparisons, for example, active control conditions (e.g., standalone media literacy or cognitive dissonance‐based training; Tobin et al. 2022), which could uncover non‐specific training effects. Future research should therefore consider including an active control group to evaluate whether BIBo effects hold. Long‐term follow‐up assessments should also be included to investigate effect stability. The generalizability of intervention effects is further impaired by BIBo's targeted design for female participants and the German study context, limiting the transferability of intervention effects to more diverse samples. Future studies could therefore include non‐binary or male participants, adolescents, individuals with different ethnic backgrounds, and individuals with varying social media use patterns.

### 4.1 Clinical Implications

BIBo holds potential to enhance the efficacy of intervention and prevention programs for EDs, buffering the effects of social media on body image. This is important because, despite prior studies finding large pre–post effects of psychotherapy on EDs, for example, for bulimia nervosa (Svaldi et al. 2019), evidence‐based ED treatment does not produce sufficient recovery rates, particularly for anorexia nervosa (Miskovic‐Wheatley et al. 2023). Given the efficacy of BIBo in the present sample, which included individuals exhibiting high ED symptoms, BIBo might also qualify as a secondary prevention tool. Furthermore, as demonstrated (Hötzel et al. 2014), online interventions can effectively increase treatment motivation. As ambivalence toward treatment is prevalent among women with EDs, especially anorexia nervosa (Ward et al. 1996), BIBo might be employed as an interim intervention prior to treatment. However, larger RCTs with long‐term follow‐up measures are needed to validate and extend the findings of the present study.

### 4.2 Conclusion

This pilot RCT assessed the efficacy of the four‐week online intervention BIBo, as a theoretically informed intervention directly addressing social comparison and ideal internalization. Overall, BIBo reduced reactivity to appearance‐related social media content, ED psychopathology, and measures of body dissatisfaction, social comparison, and thin ideal internalization in female participants with elevated ED symptoms. Results illustrate the therapeutic potential of BIBo within various intervention and prevention contexts. As a low‐threshold program, BIBo could complement ED treatment to improve outcomes.

## Author Contributions

**Gritt Ladwig:** conceptualization, investigation, writing – original draft, methodology, software, data curation. **Kristine Schönhals:** conceptualization, investigation, software, writing – review and editing. **Hannah L. Quittkat:** conceptualization, writing – review and editing. **Fanny Alexandra Dietel:** methodology, formal analysis, data curation, writing – review and editing, writing – original draft. **Silja Vocks:** conceptualization, supervision, project administration, writing – review and editing.

## Conflicts of Interest

The authors declare no conflicts of interest.

## Supporting information


**Data S1:** Supporting Information.

## Data Availability

The data that support the findings of this study are available on request from the corresponding author. The data are not publicly available due to privacy or ethical restrictions.

## Endnotes

^1^
Supplementary analyses and information on psychometric properties are accessible via our OSF repository: https://osf.io/dwfm2/.

^2^
To enhance comparability, we performed 2 × 2 mixed ANOVAs for the complete analysis and ITT. The pattern of results was replicated across all variables.
