# Supplementary material for: Addressing the Negative Impact of Social Media on Body Image: An Online Randomized Controlled Pilot Trial
Source: Int J Eat Disord. 2025 Oct 28;59(2):332–45. doi: 10.1002/eat.24584 (PMC12884261; doi:10.1002/eat.24584)
Supplement: Supplementary file 1 — Data S1: Supporting Information. [file EAT-59-332-s001.docx]

**Supplementary Materials**

**Supplement S1**

**Psychometric properties.**

Internal consistency ranges in reference to the present study are reported across time points (i.e., T0 and T1).

*Body Image States Scale (BISS).* Internal consistency in the present sample was higher than in the original study (Cronbach’s α = .77 - .90, Cash et al., 2002), with α = .88 - .92 at pre-task and α = .92 - .93 at post-task.

*Eating Disorder Examination Questionnaire – Short (EDE-QS).* The EDE-QS (Gideon et al., 2016, 2018; own translation) has shown high convergent validity with the EDE-Q (Fairburn & Beglin, 1994) for persons with and without eating disorders as well as high internal consistency (α = .91; Gideon et al., 2016. In this study, internal consistency was high (α = .74 - .80).

*Eating Disorder Inventory-2 (EDI-2).* The EDI-2 (Garner, 1991; Paul & Thiel, 2005) has been evaluated as reliable, e.g., in female outpatients with binge eating disorder (Tasca et al., 2003). Internal consistency of the Body Dissatisfaction subscale has been shown to be high (α = .89 in German women with EDs; Thiel et al., 1997. In this study, internal consistency was likewise high (α = .80 - .83).

*Upward Appearance Comparison Scale (UPACS; O’Brien et al., 2009)*. The validity and reliability of the German UPACS (Schönhals et al., 2024) have been evaluated as satisfactory, including acceptable internal consistency scores in women with EDs (McDonald’s ω_t_ = 0.93; Schönhals et al., 2024). In the present study, internal consistency was high (α = .83 - .86).

*Appearance Comparison on Social Media Scale (ACSMS).* The original ACSMS (Mahon & Hevey, 2023), which was later shortened by the authors, showed good concurrent and discriminant validity. To the best of our knowledge, to date, no internal consistency scores have been reported for the ACSMS. Internal consistency in this study was high (α = .80 - .84).

*Sociocultural Attitudes Towards Appearance Questionnaire-4-Revised (SATAQ-4R-Female).* The SATAQ-4 has been shown to be valid and reliable, with high internal consistencies for the Thin/Low body fat subscale (α = 0.89) and the Muscular subscale in a French female ED sample (α = 0.93; Rodgers et al., 2021).In the present study, internal consistency for the individual subscales of the SATAQ-4R-Female (Flechsig et al., 2025; Schaefer et al., 2017) was sufficient to high (Internalization: Muscular: α = .94 - .94, Internalization: General Attractiveness: α = .60 - .73, Internalization: Thin/Low Body Fat: α = .82 - .83).

**References**

Cash, T. F., Fleming, E. C., Alindogan, J., Steadman, L., & Whitehead, A. (2002). Beyond Body Image as a Trait: The Development and Validation of the Body Image States Scale. *Eating Disorders*, *10*(2), 103-113. <https://doi.org/10.1080/10640260290081678>

Fairburn, C. G., & Beglin, S. J. (1994). Assessment of eating disorders: Interview or self-report questionnaire? *International Journal of Eating Disorders*, *16*(4), 363-370. https://doi.org/https://doi.org/10.1002/1098-108X(199412)16:4<363::AID-EAT2260160405>3.0.CO;2-#

Flechsig, F. J., Schönhals, K., Quittkat, H. L., & Vocks, S. (2025). [Validation of the Gender-Specific German-Language Versions of the Sociocultural Attitudes Towards Appearance Questionnaire (SATAQ-4R)]. *Psychotherapie Psychosomatik Medizinische Psychologie*. https://doi.org/10.1055/a-2648-6459 (Validierung der geschlechtsspezifischen deutschsprachigen Versionen des Sociocultural Attitudes Towards Appearance Questionnaire (SATAQ-4R).)

Garner, D. M. (1991). *Eating disorder inventory-2: Professional kit*. Psychological Assessment Resources.

Gideon, N., Hawkes, N., Mond, J., Saunders, R., Tchanturia, K., & Serpell, L. (2016). Development and Psychometric Validation of the EDE-QS, a 12 Item Short Form of the Eating Disorder Examination Questionnaire (EDE-Q). *PLoS One*, *11*(5), e0152744. https://doi.org/10.1371/journal.pone.0152744

Gideon, N., Hawkes, N., Mond, J., Saunders, R., Tchanturia, K., & Serpell, L. (2018). Correction: Development and Psychometric Validation of the EDE-QS, a 12 Item Short Form of the Eating Disorder Examination Questionnaire (EDE-Q). *PLoS One*, *13*(11), e0207256. https://doi.org/10.1371/journal.pone.0207256

Mahon, C., & Hevey, D. (2023). Pilot trial of a self-compassion intervention to address adolescents’ social media-related body image concerns. *Clinical Child Psychology and Psychiatry*, *28*(1), 307-322. https://doi.org/10.1177/13591045221099215

O'Brien, K. S., Caputi, P., Minto, R., Peoples, G., Hooper, C., Kell, S., & Sawley, E. (2009). Upward and downward physical appearance comparisons: development of scales and examination of predictive qualities. *Body Image*, *6*(3), 201-206. https://doi.org/10.1016/j.bodyim.2009.03.003

Paul, T., & Thiel, A. (2005). *Eating disorder inventory 2: EDI-2; manual*. Hogrefe.

Rodgers, R. R. F., Schaefer, L. M., Seneque, M., Alacreu-Crespo, A., Moreno-Padilla, M., Courtet, P., Thompson, J. K., & Guillaume, F. L. S. (2021). Sociocultural Attitudes Towards Appearance Questionnaire-4: Psychometric properties among a French clinical eating disorder sample and normative comparisons. *Eating Behaviors*, *40*, 101466. https://doi.org/https://doi.org/10.1016/j.eatbeh.2020.101466

Schaefer, L. M., Harriger, J. A., Heinberg, L. J., Soderberg, T., & Kevin Thompson, J. (2017). Development and validation of the sociocultural attitudes towards appearance questionnaire‐4‐revised (SATAQ‐4R). *International Journal of Eating Disorders*, *50*(2), 104-117. https://doi.org/10.1002/eat.22590

Schönhals, K., Quittkat, H. L., Voges, M. M., Ladwig, G., Holtmann, F. J., & Vocks, S. (2024). Is my body better than yours? Validation of the German version of the Upward and Downward Physical Appearance Comparison Scales in individuals with and without eating disorders. *Frontiers in Psychology*, *15*, 1390063. https://doi.org/10.3389/fpsyg.2024.1390063

Tasca, G. A., Illing, V., Lybanon-Daigle, V., Bissada, H., & Balfour, L. (2003). Psychometric Properties of the Eating Disorders Inventory-2 among Women Seeking Treatment for Binge Eating Disorder. *Assessment*, *10*(3), 228-236. https://doi.org/10.1177/1073191103255001

Thiel, A., Jacobi, C., Horstmann, S., Paul, T., Nutzinger, D. O., & Schüssler, G. (1997). A German version of the Eating Disorder Inventory EDI-2. *Psychotherapie Psychosomatik Medizinische Psychologie*, *47*(9-10), 365-376. (Eine deutschsprachige Version des Eating Disorder Inventory EDI-2.)

**Supplement S2**

*Outcome of the intention-to-treat analysis (N = 157).*

**Primary outcome measures.**

*Reactivity to appearance-related social media content.* Regarding change scores on the BISS,
there was a significant time × group interaction (*b* = 0.44, SE = 0.22, *t* = 2.05, 95% CI [0.02, 0.87], *p* = .041).
Within-group contrasts showed a significant decrease from T0 to T1 in the BIBo group (*b* = 0.46, SE = 0.16, *t* = 2.95, *p* = .004, *d* = 0.55), while the WLC showed no change (*b* = 0.02, SE = 0.15, *t* = 0.13, *p* = .898, *d* = 0.03). However, at T1, there was no significant between-group difference (*b* = –0.34, SE = 0.19, t = –1.81, p = .072, *d* = 0.40).

*Eating disorder symptoms.* Regarding the EDE-QS, a significant time × group interaction emerged (*b* = 4.33, SE = 0.93, *t* = 4.68, 95% CI [2.52, 6.15], *p* < .001).
Within-group contrasts showed that the BIBo group decreased significantly from T0 to T1 (*b* = 4.65, SE = 0.68, *t* = 6.85, *p* < .001, *d* = 0.71), while the WLC did not (*b* = 0.32, SE = 0.63, *t* = 0.50, *p* = .619, *d* = 0.06). At T1, the BIBo group scored significantly lower than the control group (*b* = –3.88, SE = 1.09, t = –3.56, p = .001, *d* = 0.50).

**Secondary outcome measures.**

*Social comparison.* Regarding the UPACS, we found a significant time × group interaction (*b* = 0.37, SE = 0.10, *t* = 3.84, 95% CI [0.18, 0.55], *p* < .001).
Within-group contrasts showed a significant reduction in the BIBo group (*b* = 0.35, SE = 0.07, *t* = 5.04, *p* < .001, *d* = 0.58), while no change was observed in the WLC (*b* = –0.01, SE = 0.07, *t* = –0.23, *p* = .822, *d* = 0.01).
Further, at T1, the BIBo group scored significantly lower than the WLC (*b* = –0.28, SE = 0.11, *t* = –2.58, *p* = .011, *d* = 0.31).

*Appearance comparison on social media.* Regarding the ACSMS, there was a significant time × group interaction (*b* = 3.79, SE = 0.93, *t* = 4.07, 95% CI [1.96, 5.61], *p* < .001).
Within-group contrasts showed a significant decrease in the BIBo group (*b* = 4.61, SE = 0.68, *t* = 6.75, *p* < .001, *d* = 0.88), while the WLC demonstrated no significant change (*b* = 0.82, SE = 0.63, *t* = 1.29, *p* = .199, *d* = 0.08). Further, at T1, the BIBo group scored significantly lower than the WLC (*b* = –4.35, SE = 1.12, *t* = –3.90, *p* < .001, *d* = 0.58).

*Body dissatisfaction.* Regarding the EDI-2 BD, we found a significant time × group interaction (*b* = 2.69, SE = 0.83, *t* = 3.26, 95% CI [1.07, 4.30], *p* = .001).
Within-group contrasts showed a significant decrease in the BIBo group (*b* = 2.66, SE = 0.61, *t* = 4.38, *p* < .001, *d* = 0.38), while the WLC showed no change (*b* = –0.03, SE = 0.56, *t* = –0.06, *p* = .952, *d* = 0.02). At T1, the BIBo group scored significantly lower than the WLC (*b* = –3.08, SE = 1.23, *t* = –2.50, *p* = .013, *d* = 0.11).

*Body ideal internalization.* Regarding the SATAQ-4R subscales, for thin ideal internalization, a significant time × group interaction emerged (*b* = 0.39, SE = 0.13, *t* = 3.07, 95% CI [0.14, 0.63], *p* = .003).
Follow-up within-group contrasts showed that the BIBo group decreased significantly from T0 to T1 (*b* = 0.33, SE = 0.09, *t* = 3.54, *p* < .001, *d* = 0.41), whereas the WLC did not (*b* = –0.06, SE = 0.09, *t* = –0.69, *p* = .490, *d* = 0.08). However, at T1, the groups did not differ significantly (*b* = –0.07, SE = 0.16, t = –0.42, p = .672, *d* = 0.06).
For the muscular and attractiveness ideal internalization, the time × group interactions were non-significant (muscular ideal: *b* = 0.07, SE = 0.16, *t* = 0.46, 95% CI [–0.24, 0.39], *p* = .649; attractiveness ideal: *b* = 0.13, SE = 0.09, *t* = 1.41, 95% CI [–0.05, 0.30], *p* = .160).
